# Supplementary material for: Veterinary consumption of highest priority critically important antimicrobials and various growth promoters based on import data in Pakistan
Source: PLoS One. 2022 Sep 14;17(9):e0273821. doi: 10.1371/journal.pone.0273821 (PMC9473402; doi:10.1371/journal.pone.0273821)
Supplement: S1 File — (PDF) [file pone.0273821.s001.pdf]

Supplementary Material

Veterinary Consumption of Highest Priority Critically Important Antimicrobials and Various Growth Promoters based on Import Data in Pakistan

Table S1: Import volumes of different antimicrobial feed additive/growth promoter products for the years 2017-19.

**Antimicrobial feed additives**	**AAI strength**	**Tonnes imported**			
		**2017**	**2018**	**2019**	**Total**
Zinc Bacitracin		1036.73	344.16	636.37	2017.26
	Zinc Bacitracin 15%	999.44	325.1	625.93	
	Zinc Bacitracin 10%	37.29	19.06	10.44	
Enramycin	Enramycin 4%	375.41	757.12	432.08	1564.61
Colistin Sulphate		191.82	144.32	188.06	524.2
	Colistin 10%	72.82	28.32	93.06	
	Colistin 0.294%[1]	64	88.5	95	
	Colistin 0.06%[2]	55	27.5	-	
Bacitracin Methylene Disalicylate (BMD)		62.94	103.47	220.12	386.53
	BMD 15%	23.94	94.02	214.67	
	BMD 10%	39	9.45	5.45	
Tylosin Phosphate / Tylosin Tartrate		132.44	78.94	83.28	294.66
	Tylosin 25%	37.34	13.05	24.39	
	Tylosin 10%	95.1	65.89	58.89	
Lincomycin Hydrochloride		92.19	77.96	113.55	283.7
	Lincomycin 11%	38.64	30.25	35.91	
	Lincomycin 4.4%	53.55	37.71	77.64	
	Lincomycin 1.1%	-	10	-	
Tiamulin Hydrogen Fumarate		52.78	40.96	35.35	129.09
	Tiamulin 98%	8	9.92	4.35	
	Tiamulin 10%	44.78	31.04	31	
Flavophospholipol		0	16.28	76.5	92.78
	Flavophospholipol 40%	-	6.78	-	
	Flavophospholipol 8%	-	9.5	76.5	
Tilmicosin	Tilmicosin 25%	-	-	20	20
Total		1944.31	1563.21	1805.31	5312.83

[1,2]Products containing other antimicrobials in addition to colistin sulphate i.e., procaine penicillin 1.2%, streptomycin sulphate 3.6%, and zinc bacitracin 5.2%.

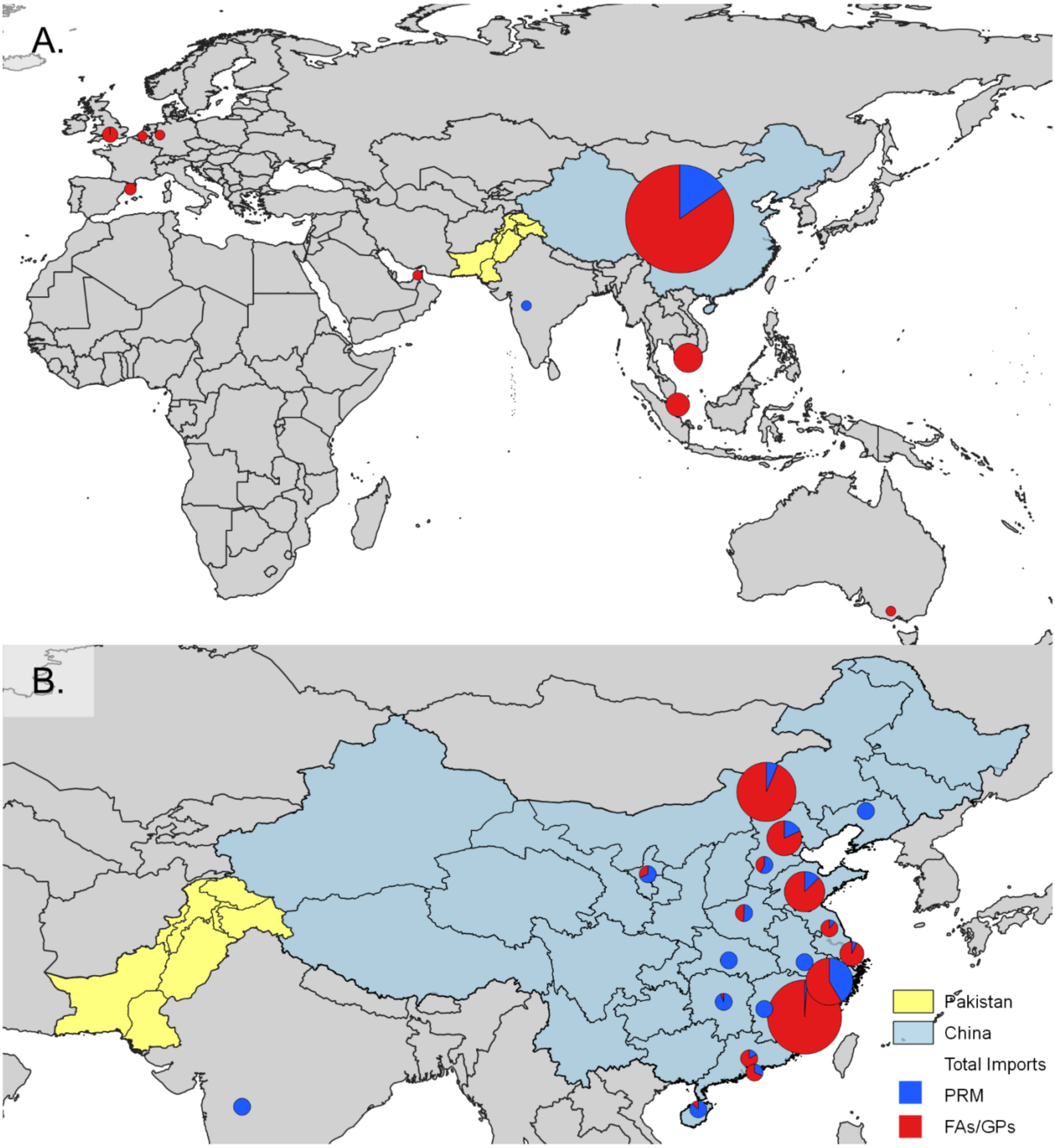

Figure S1: Total volumes and ratios of pharmaceutical raw materials and feed additive/growth promoter products for the years 2017-19 imported from: A. different countries and B. China and its different provinces.

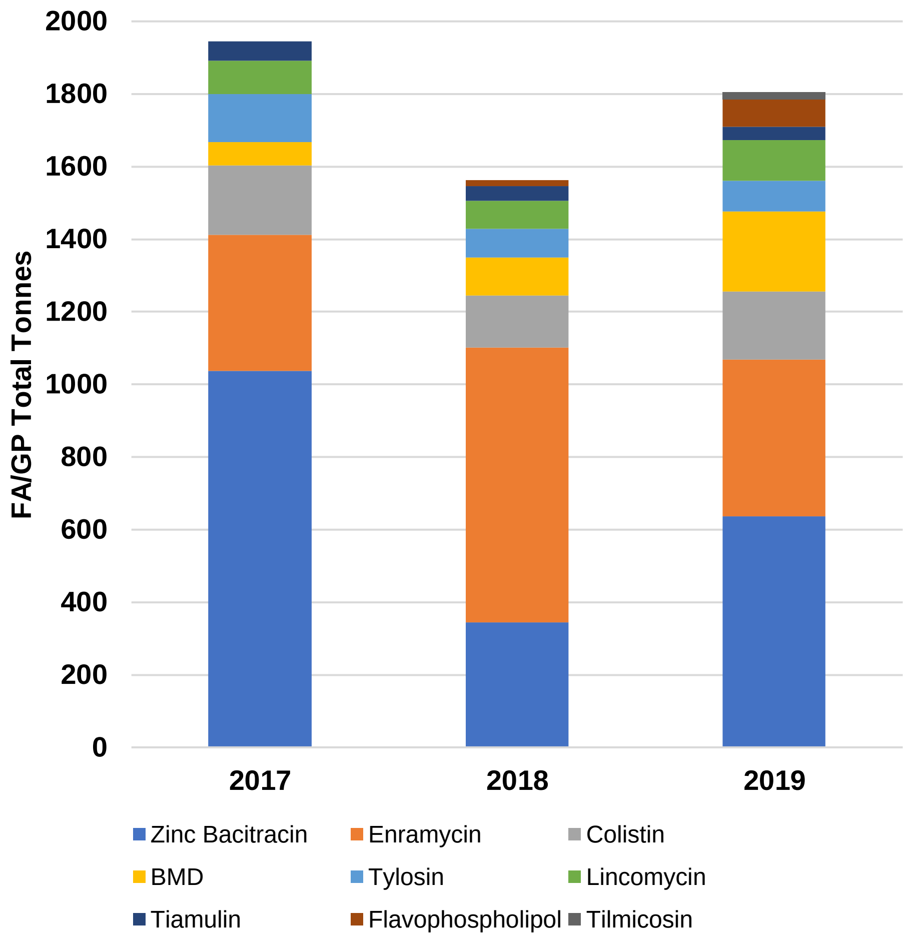

Figure S2: Total import volumes of antimicrobial veterinary feed additive/growth promoter products for the years 2017-19. For small amounts imported referred to Table S1.

Table S2: Total animal biomass calculation for the species likely to be treated with antimicrobials for the years 2017-19.

Species	Year	Live Animals[1] (000 heads)	Data Source[2]	Animals slaughtered (000 heads)	Meat production (000 tonnes)	Carcass weight[3] (kg)	Conversion coefficient[4] (K)	Liveweight[5] (kg)	Animal biomass[6] (000 tonnes)
Buffaloes	2019	40002	Pakistan	7647	1085	141.89	0.56	253.38	8059
Cattle	2019	47821	Pakistan	8728	1136	130.16	0.56	232.43	9186
Goats	2019	76143	Pakistan	41915	491	11.71	0.47	24.91	4659
Sheep	2019	30859	Pakistan	14810	241	16.27	0.47	34.62	2087
Chickens	2019	1321000	Pakistan	1278000	1518	1.19	0.7	1.7	2173
Camels	2019	1090	Asia	1159.126	230	198.44	0.55	360.8	393
Asses	2019	5417	Asia	1663.167	143	86.14	0.5	172.28	933
Mules	2019	196	Asia	130.184	15	116.86	0.5	233.72	46
Horses	2019	371	Asia	3094.729	430	138.85	0.5	277.7	103
Buffaloes	2018	38848	Pakistan	7426	1054	141.93	0.56	253.45	7829
Cattle	2018	46084	Pakistan	8411	1095	130.19	0.56	232.48	8854
Goats	2018	74134	Pakistan	40810	478	11.71	0.47	24.91	4536
Sheep	2018	30498	Pakistan	14637	239	16.33	0.47	34.74	2064
Chickens	2018	1210000	Pakistan	1167000	1391	1.19	0.7	1.7	1984
Camels	2018	1076	Asia	1100.833	219	198.63	0.55	361.15	389
Asses	2018	5319	Asia	1566.291	134	85.82	0.5	171.64	913
Mules	2018	192	Asia	150.067	17	116.03	0.5	232.06	45
Horses	2018	369	Asia	2912.889	396	135.83	0.5	271.66	100
Buffaloes	2017	37700	Pakistan	7212	1024	141.99	0.56	253.55	7601
Cattle	2017	44400	Pakistan	8106	1055	130.15	0.56	232.41	8528
Goats	2017	72179	Pakistan	39733	465	11.7	0.47	24.89	4416
Sheep	2017	30141	Pakistan	14466	236	16.31	0.47	34.7	2039
Chickens	2017	1108000	Pakistan	1066000	1276	1.2	0.7	1.71	1823
Camels	2017	1062	Asia	1048.964	209	199.14	0.55	362.07	385
Asses	2017	5222	Asia	1599.445	136	85.15	0.5	170.3	889
Mules	2017	189	Asia	158.299	18	114.1	0.5	228.2	43
Horses	2017	367	Asia	2663.448	362	135.77	0.5	271.54	100

[1]FAOSTAT live animals' data for Pakistan.
[2]FAOSTAT data source for animals slaughtered and meat production.
[3]Carcass weight = Meat production ÷ Animals slaughtered, WOAH methodology [1].
[4]Conversion coefficients (K) taken from "Manual for the compilation of supply balance sheets for meat" [2]. Whereas for camel K value is taken from "Camel meat and meat products" [3].
[5]Live weight = Carcass weight ÷ Conversion coefficient (K), WOAH methodology [1].
[6]Total animal biomass values for 2017, 2018, and 2019 were 25822733.05, 26713113.38, and 27638499.83 tonnes whereas for chicken biomass values were 1822860, 1983900, and 2172600 tonnes, respectively. The average total biomass for the years 2017-19 was 26724782.09 tonnes.

Calculation of biomass

Biomass of different animal species was calculated using the following equations (**Eq S1-4)** [1].

$$\boldsymbol{Biomass}\ (\boldsymbol{buffaloes\ and\ cattle}) = Live\ animals \times [(Live\ weight \times LSU_{calves} \times P.pop_{calves}) + (Live\ weight \times LSU_{young} \times P.pop_{young}) + (Live\ weight \times LSU_{adults} \times P.pop_{adults})] \quad \textbf{Eq. S1}$$

For buffaloes and cattle population proportions (P.pop) for calves (<1 years), young (1-3 years), and adult (>3 years) were calculated from Pakistan Livestock Census 2006 [4]. Calves, young, and adults P.pop for buffaloes were 29.36%, 11.48%, 59.16% whereas for cattle were 24.82%, 9.87%, 65.31%, respectively. Livestock unit ratios (LSU) for calves, young, and adult were taken as 0.4, 0.75, and 1, respectively [5].

$$\boldsymbol{Biomass}\ (\boldsymbol{goats\ and\ sheep}) = (Animals\ slaughtered \times Live\ weight) + \left[Live\ animals - \left(\frac{Animals\ slaughtered}{1.5}\right) \times 75kg\right] \quad \textbf{Eq. S2}$$

$$\boldsymbol{Biomass}\ (\boldsymbol{chicken}) = Animals\ slaughtered \times Live\ weight \quad \textbf{Eq. S3}$$

$$\boldsymbol{Biomass}\ (\boldsymbol{camels, asses, mules, and\ horses}) = Live\ animals \times Live\ weight \quad \textbf{Eq. S4}$$

Table S3: Antimicrobial active ingredients and their chemical compounds imported as pharmaceutical raw materials.

Antimicrobial Active Ingredients (AAI)	Antimicrobial Chemical Compound (ACC)
Tylosin	Tylosin Tartrate / Tylosin Phosphate
Enrofloxacin	Enrofloxacin Base
Colistin	Colistin Sulphate
Tilmicosin	Tilmicosin Phosphate
Spiramycin	Spiramycin Adipate
Norfloxacin	Norfloxacin Base
Ceftiofur	Ceftiofur Sodium / Ceftiofur Hydrochloride

Table S4: Antimicrobial active ingredients and their chemical compounds for the products imported as feed additives/growth promoters.

Antimicrobial Active Ingredients (AAI)	Antimicrobial Chemical Compound (ACC)
Zinc Bacitracin	Zinc Bacitracin
Enramycin	Enramycin
BMD	Bacitracin Methylene Disalicylate (BMD)
Tylosin	Tylosin Phosphate
Colistin	Colistin Sulphate
Lincomycin	Lincomycin Hydrochloride
Tiamulin	Tiamulin Hydrogen Fumarate
Flavophospholipol	Flavophospholipol
Tilmicosin	Tilmicosin
Penicillin	Procaine Penicillin
Streptomycin	Streptomycin Sulphate

References

1. Góchez D, Raicek M, Pinto Ferreira J, Jeannin M, Moulin G, Erlacher-Vindel E. OIE Annual Report on Antimicrobial Agents Intended for Use in Animals: Methods Used. Frontiers in Veterinary Science. 2019;6: 317. doi:10.3389/fvets.2019.00317
2. Eurostat. Manual for the Compilation of Supply Balance Sheets for Meat. 2009 [cited 21 Aug 2022]. Available: https://circabc.europa.eu/sd/a/90447c6f-5b7c-4b6f-87e9-27c5a7a5c923/ASA-TE-F-655%2520SBS%2520Manual%2520-%2520meat.doc
3. Kadim IT, Mahgoub O, Faye B, Farouk M, editors. Camel meat and meat products. CABI; 2013.
4. GOP. Pakistan Livestock Census 2006. 2006 [cited 21 Aug 2022]. Available: https://www.pbs.gov.pk/publication/pakistan-livestock-census-2006
5. Eurostat. Glossary: Livestock unit (LSU). 2020 [cited 21 Aug 2022]. Available: https://ec.europa.eu/eurostat/statistics-explained/index.php?title=Glossary:Livestock_unit_(LSU)
