# Supplementary material for: Veterinary consumption of highest priority critically important antimicrobials and various growth promoters based on import data in Pakistan
Source: PLoS One. 2022 Sep 14;17(9):e0273821. doi: 10.1371/journal.pone.0273821 (PMC9473402; doi:10.1371/journal.pone.0273821)
Supplement: Attachment — Submitted filename: Response to Editor and Reviewers.docx [file pone.0273821.s003.docx]

**1^st^ Response to Editor**

1. Please ensure that your manuscript meets PLOS ONE's style requirements, including those for file naming. The PLOS ONE style templates can be found at https://journals.plos.org/plosone/s/file?id=wjVg/PLOSOne_formatting_sample_main_body.pdf and https://journals.plos.org/plosone/s/file?id=ba62/PLOSOne_formatting_sample_title_authors_affiliations.pdf

**RESPONSE: We have adjusted the manuscript according to the style requirements.**

2. Please provide additional details regarding participant consent. In the ethics statement in the Methods and online submission information, please ensure that you have specified (1) whether consent was informed and (2) what type you obtained (for instance, written or verbal, and if verbal, how it was documented and witnessed). If your study included minors, state whether you obtained consent from parents or guardians. If the need for consent was waived by the ethics committee, please include this information.

If you are reporting a retrospective study of medical records or archived samples, please ensure that you have discussed whether all data were fully anonymized before you accessed them and/or whether the IRB or ethics committee waived the requirement for informed consent. If patients provided informed written consent to have data from their medical records used in research, please include this information.

**RESPONSE: Not applicable as this is not a human subject study.**

3. We note that Figures 1 and S1 in your submission contain map images which may be copyrighted. All PLOS content is published under the Creative Commons Attribution License (CC BY 4.0), which means that the manuscript, images, and Supporting Information files will be freely available online, and any third party is permitted to access, download, copy, distribute, and use these materials in any way, even commercially, with proper attribution. For these reasons, we cannot publish previously copyrighted maps or satellite images created using proprietary data, such as Google software (Google Maps, Street View, and Earth). For more information, see our copyright guidelines: http://journals.plos.org/plosone/s/licenses-and-copyright.

We require you to either (1) present written permission from the copyright holder to publish these figures specifically under the CC BY 4.0 license, or (2) remove the figures from your submission:

a. You may seek permission from the original copyright holder ………………….

**RESPONSE: Figures 1 and S1 (choropleth maps) were developed in QGIS Desktop 3.22.3 (a free and open-source geographic information system** [**https://qgis.org/en/site/**](https://qgis.org/en/site/)**). The shape files (.shp) for the development of Figures 1 and S1 were sourced from DIVA-GIS (**[**https://www.diva-gis.org/gdata**](https://www.diva-gis.org/gdata)**) free spatial data (**[**https://www.diva-gis.org/docs/DIVA-GIS_manual_7.pdf**](https://www.diva-gis.org/docs/DIVA-GIS_manual_7.pdf)**). However, spatial map for Pakistan was developed by the authors using Adobe Illustrator and QGIS. Moreover, the data for the development of choropleth was sourced from PakExim Trade Info as described in methods section and provided as metadata.**

**The authors claim the ownership of Figures 1 and S1 choropleth maps.**

4. Please include captions for your Supporting Information files at the end of your manuscript, and update any in-text citations to match accordingly. Please see our Supporting Information guidelines for more information: http://journals.plos.org/plosone/s/supporting-information.

**RESPONSE: Captions added at the end of manuscript.**

5. Please upload a copy of Supporting Information table S3 and S4 which you refer to in your text on page 8.

**RESPONSE: All the supplementary tables and figures, including tables S3 and S4 were included in Supplementary Material file uploaded along with the submission files.**

**2^nd^ Response to Editor**

1. We notice that your manuscript file was uploaded on December 21, 2021. Please can you upload the latest version of your revised manuscript as the main article file, ensuring that does not contain any tracked changes or highlighting.  This will be used in the production process if your manuscript is accepted. Please follow this link for more information: [http://blogs.PLOS.org/everyone/2011/05/10/how-to-submit-your-revised-manuscript/](http://blogs.plos.org/everyone/2011/05/10/how-to-submit-your-revised-manuscript/)

**RESPONSE: We have uploaded the latest clean version of our revised manuscript as Main File in addition to tracked changes version. As per above blog link, original manuscript file of December 21, 2021 is also retained during submission.**

2. Please note that PLOS utilizes the CC BY 4.0 license (<https://creativecommons.org/licenses/by/4.0/>) which means that all material on our website is freely available online, and any third party is permitted to access, download, copy, distribute, and use these materials in any way, even commercially, with proper attribution. If the figures have already been published, authors must provide proper attribution, referencing the source clearly, and obtain permissions if the image is copyrighted.
Before we can proceed with your manuscript, can you please clarify the source of the Pakistan Exim Trade Info that was used to create the maps in Fig 1 and Fig S1? The URL you provided in the citation, "<https://pak.eximtradeinfo.com/,>" does not work and turns up no results when we search for it.

**RESPONSE: Kindly note that all the materials/source files are already available in the supplementary metadata within our submission. In addition, all the images are original and not copyrighted.**

**Please note that we subscribed** [**https://pak.eximtradeinfo.com**](https://pak.eximtradeinfo.com) **database and website was assessed until 30 July 2021 for our import data source files. Currently the website is not functioning. However, all the source files have been attached in the metadata.**

**In this aspect changes are made in Lines 119-121: We used the Pakistan Exim Trade Info database as our import data source. This commercially available database contained all imports and exports of goods to and from Pakistan and was accessible until July 2021 [14].**

**Response to Reviewer 1**

Overall impression

This was a decent attempt at quantifying antimicrobial imports in Pakistan focusing on selected CIA-HtPs and feed additives/growth promoters. The Methods and Result section are well written and transparent although small sections of the methodology require amendments to improve clarity. Equations 1 to 6 are outside of the peer-reviewer’s expertise to comment.

**RESPONSE: We thank the reviewer for positive comments. Changes are incorporated into methods as suggested by the reviewer.**

The Discussion and Conclusion section requires substantial development and redrafting before publication. There are several sweeping and unsubstantiated statements which requires referencing (e.g. line 237-238) or elaboration (e.g. line 274-275). The study also needs a deeper technical interpretation of the findings and the findings need to be linked to other relevant research, even if it is outside of Pakistan. Several key points can be better articulated and developed (e.g. Line 190-192; Line 260-268).

**RESPONSE: Thank you for these observations. We have addressed these comments point-by-point.**

Finally, there are grammatical errors that need to be addressed to improve readability, especially in the Discussion section.

**RESPONSE: Grammatical errors have been rechecked and corrected.**

Discussion of specific areas for improvement (Major issues)

• Line 99 and Line 300-301: It should be clarified why CIA-HtPs were only identified on dairy and broiler farms but not on other types of farms. This is especially important since other species of animals (e.g. buffalos, goats and sheep) contribute to a significant portion of total animal biomass. See Figure 4 in manuscript. In line 301, explain to the audience why it is a reasonable assumption that the 7 CIA-HtPs were the most frequent ones used on farms.

**RESPONSE: In Pakistan commercial diary is referred to both the buffalo and cattle farming (**[**https://www.intechopen.com/chapters/65652**](https://www.intechopen.com/chapters/65652)**). In the survey dairy farms, both buffalo and cattle were reared. In Pakistan, antimicrobials are frequently used in commercial intensive-livestock farming like poultry and dairy (**[**https://pubmed.ncbi.nlm.nih.gov/31795863/**](https://pubmed.ncbi.nlm.nih.gov/31795863/) **;** [**https://pubmed.ncbi.nlm.nih.gov/33324699/**](https://pubmed.ncbi.nlm.nih.gov/33324699/) **;** [**https://pubmed.ncbi.nlm.nih.gov/34069928/**](https://pubmed.ncbi.nlm.nih.gov/34069928/)**). Contrary, sheep, goats and other animals are reared on open grazing lands, and we assumed less antimicrobial usage in these animal species. Moreover, the antimicrobials formulations for large (Bovines, Equine, and Camelids) and small animals (Caprine and Ovine) are the same with dosage instructions printed on brand label/package, but different for those used in poultry. Survey of broiler and dairy farms was used as proxy to look into the brands and antimicrobials used in food animals.**

**Lines 348-351 modified: “There may be more critically important antimicrobials used on Pakistani farms; however, based on the present survey and previously published studies [8–10] we reasonably assume that the seven we included in our study are the most frequently used ones.”**

• Line 173: It should be explained why only total chicken biomass was used. Perhaps total broiler biomass should be used instead, since the growth promoters and feed additives were only used on broilers.

**RESPONSE: Thank you for this comment. We added the lines 173-176 “It is important to note that these FAs/GPs were exclusively imported to be used in poultry feed as evident from the importers that are poultry feed manufacturers or suppliers for the poultry feed. Pakistan’s total poultry counts show 95% (1279.76/1353.24 Million no.) of the broiler population compared to 4% (59.82/1353.24 Million no.) of the layers in 2019-20** [**https://www.finance.gov.pk/survey/chapter_20/PES_2019_20.pdf**](https://www.finance.gov.pk/survey/chapter_20/PES_2019_20.pdf)**.”**

**Moreover, the feed mills use feed additives/growth promoters aiming to enhance the quality of their feed irrespective of the type of feed i.e., for layers and boilers. Hence, we use the term total chicken biomass.**

• Line 190-192: An effort could be made to discuss the origin of antimicrobials into Pakistan in the Discussion section. For example, if these exporting locations were the country of manufacture or transit.

**RESPONSE: These locations refered to manufacturing/exporting places not transit. Referred to methodology line 133-133 “The countries exporting PRMs and FAs/GPs to Pakistan were determined by online searching the exporting company country of origin.”. However, a section in discussion is added for further details.**

**Lines 128-132 added: “Information on each shipment can be categorized into three groups i) importer and exporter details, ii) details on commodities, and iii) details on shipment date, identity, and shipping route. Exporting countries were identified based on the exporter/manufacturer details and not on the vessel departure port which may mislead to the transit countries.”**

• Line 237-238: This statement requires substantiation with a reference. Studies that have shown a decline (instead of increase) in the use of medically important antimicrobials in food animals should also be considered.

**RESPONSE: We thank the reviewer for this comment. We agree and made the following modifications:**

**Lines 258-269 added: “Advances in collective knowledge of the dynamics between antimicrobial use and resistance has promoted the need for surveillance, with this information applied to policies and practices aimed as AMR reduction. For example, the European Union, leaders in recognition of the link between use and resistance, have long-running surveillance systems to inform both policy and practice. For example, the European Surveillance for Antimicrobials Consumption (ESVAC) reported an overall decrease in the sales of veterinary antimicrobials in 31 European countries between from 2010 to 2018. The decrease was significantly impacted by the decline in sales of antimicrobials with high risk to public health [4]. A similar decreasing trend was reported from other high-income countries and some upper-middle income countries such as China which is attributed to effective AMU stewardship efforts [21]. Thus, routine collection, and use, of antimicrobial consumption data, as these examples suggest, is an important tool to promote public health, with regards to AMR containment.”**

**Lines 270-276 modified: “Contrastingly to findings from HIC where the use of medically important antimicrobials in food animals are declining, we found in Pakistan no decrease in the import of these antimicrobials along with the growth promoters in the study period 2017-2019.”**

• Line240: This should be PRM. See Table 2 and line 181.

**RESPONSE: Line 279: Correction made.**

• Line 245: Suggestion removal of the word “companies”, as the main manuscript does not include any description on companies exporting growth promoters in the veterinary sector.

**RESPONSE: Line 286: Suggestion accepted.**

• Line 260-268: It seems that the main point of this paragraph is that there is a high level of use of CIAs in animals in Pakistan. This was not clear from the first sentence in line 260. In addition, line 161 and 162 could be further developed in terms of comparing Pakistan’s level of CIA use in animals to other countries with a similar agriculture system.

**RESPONSE: We agree with the reviewer. Line 303 was modified: “Second, our study provides an estimate on the high-level consumption of CIA with highest priority.”**

**There is a lack country level data on veterinary consumption of antimicrobials from LMICs (**[**https://www.mdpi.com/2079-6382/7/3/75**](https://www.mdpi.com/2079-6382/7/3/75)**) and countries with similar agricultural system as of Pakistan. Moreover, the difference in methodologies makes comparison difficult among the available data as mentioned in Lines 338-341 “It is difficult to compare the veterinary antimicrobial consumption per unit biomass due to lack of any standardized metric and monitoring system [30]. Moreover, our findings are difficult to be compared with other AMU studies because we focused only on critically important antimicrobials and not all classes of antimicrobials.”**

• Line 272: This statement regarding the lack of serious attention in Pakistan on the need for better regulation and enforcement requires substantiation or a reference.

**RESPONSE: We agree with the reviewer and added a reference in Line 317-318:** [**https://www.thelancet.com/journals/laninf/article/PIIS1473-3099(18)30516-4/fulltext**](https://www.thelancet.com/journals/laninf/article/PIIS1473-3099(18)30516-4/fulltext)

• Line 272-273: The point that feed additives were used exclusively in broilers should be mentioned in the “Results” section.

**RESPONSE: We have revised these lines as per reviewer above-mentioned point. Lines 211-213 added in results: “These FAs/GPs were exclusively imported to be used in poultry feed as evident from the importers that are poultry feed manufacturers or their suppliers (Supplementary Metadata, Figure S1).”**

• Line 274:275: There is a leap of logic in this statement. High use of antimicrobials not mean that there will be high residue levels in the meat, as long as withdrawal periods are adhered to. Residues in milk can also be discussed since it is a large industry in Pakistan.

**RESPONSE: We agree with the reviewer and deleted Line 320-321.**

• Line 279: It is unclear where 175 tonnes per year was obtained from as this value was not mentioned in the manuscript. Also clarify if this numbers refers to PRM of the 7 CIA-HtP, AAI of chicken feed additive/growth promoters or both.

**RESPONSE: Line 324-325 modified: Here, we found colistin based FAs/GPs product import estimating approximately 175 tonnes per year (524.2 tonnes during 2017-19) (Table S1).**

• “Limitations” section (line 294-309): The inclusion of only PRMs (and hence exclusion of active pharmaceutical ingredient and finished pharmaceutical products) for the 7 CIA-HtPs is a limitation of this study – this was not mentioned. Separately, the authors could comment on the impact of using PRM to estimate import volume, and why they were not converted to AAI.

**RESPONSE:**

|  | PRM Pharmaceutical Raw Material | ACC Antimicrobial Chemical Compound OR Active Pharmaceutical Compound | AAI Antimicrobial Active Ingredient OR Active Moiety |
| --- | --- | --- | --- |
| Example | Tylosin Tartrate, Colistin Sulphate | Tylosin Tartrate, Colistin Sulphate | Tylosin, Colistin |
| In Paper | Used for Critically Important Antimicrobials with Highest Priority (CIA-HtP) imports and calculations | Used for calculation of Feed Additives/Growth Promoters (FAs/GPs) products | Not used  AAI changed to ACC |

**In this study PRM represents the Active Pharmaceutical Ingredient (API). However, the calculations are made on Actual Compound not the Active Moiety, hence represented as PRM and not as AAI OR API. Considering this facts authors changed the AAI (used to represent the chemical compound in FAs/GPs products) to ACC. Moreover, the OIE** [**https://www.frontiersin.org/articles/10.3389/fvets.2019.00317/full**](https://www.frontiersin.org/articles/10.3389/fvets.2019.00317/full) **accepts national level reporting of AMC data based on ACC.**

**Is this respect we made the following changes in the manuscript:**

**Line 160-161 modified in methodology: “All the net weight calculations for the 7 different CIA-HtPs were made against their chemical compounds given in Table S3-4 [6]”**

**Line 353-354 added in limitations: “The calculations were based on PRM or the ACC of active ingredient (moiety) however, OIE accepts AMC reporting based on the amount of ACC [6].”**

**We agree with the reviewer this study excluded the Finished Pharmaceutical Products, for this limitation and possible explanation was mentioned in Lines 356-359.**

• The Abstract should not contain information that was not mentioned in the main manuscript (e.g. Watch or Reserve list by WHO) Discussion of specific areas for improvement (Minor issues)

**RESPONSE: We agree with the reviewer and added Lines 305-307 in discussion: “These antimicrobials belong to macrolides, quinolones, polymyxins and cephalosporins listed in Watch and Reserve categories of WHO AWaRE classification indicating antimicrobials with higher resistance potential and reserved for life multi-drug resistant infections.”**

• Grammar issues (e.g. Line 34, 244, 245-247, line 288, line 306-307)

**RESPONSE: Line 34-35 modified: “…… are some also on the Watch or Reserve list by the WHO, indicating the need for stewardship and to conserve essential antimicrobials to contain AMR.”**

**Line 285-286 modified: “…… describing import quantities, consumption, and details on exporting countries ……”**

**Line 284-286 modified: “Our study fills an important knowledge gap by describing import quantities, consumption, and details on exporting countries of growth promotors in the veterinary sector”**

**Line 334-335 needful done: “When considering import volumes and subsequent use of growth promoters in chicken zinc bacitracin ……….”**

**Line 356-357 correction made: “We assume that Pakistan imports of finished veterinary antimicrobial products”**

• Line 58: OIE has introduced a method to collect national-level consumption data which adjusts for biomass. The template does not adjust for biomass.

**RESPONSE: Lines 60-64 modified: “In this context, OIE published different templates to collect national-level animals AMC data based on the amounts of antimicrobial classes used or sold for medical use and growth promotion further stratifying by different animals and administration routes.”**

• Line 73: Please clarify if there are very few companies manufacturing AAI, or a very low volume of AAIs being manufactured by companies.

**RESPONSE: Thank you for this comment. Only very few companies manufacture pharmaceutical raw material in Pakistan in a very low volume.**

**Lines 78-83 modified: “According to industry reports, there is low local manufacturing of pharmaceutical raw material (PRM), with almost 95% of the PRM is imported in Pakistan to be processed for product manufacturing by the human and veterinary pharmaceutical manufacturing industry [12,13].”**

• Line 79: Normalization is a statistical term which has not been used in this field. OIE uses the term adjusted. (i.e. adjusted for animal biomass). This applies to the rest of the manuscript which uses the term “normalization”.

**RESPONSE: Line 86 accepted and changed: “Normalization” change to “Adjusted for”. Similar done in the rest of manuscript.**

• Line 104: Please clarify the statement “questionnaires were recollected as photographs…”. Recollect might not be a appropriate term here.

**RESPONSE: Line 111 accepted and changed: “Recollected” changed to “Received”. And Line 111-112 modified: Questionnaires were received as photographs or images (digital/soft copies) or as hard form (filled-in paper copies) via postal mail.**

• Line 243: The authors could be more specific here. Is farming more intensive in Pakistan?

**RESPONSE: The authors mentioned animal population and farming type in Line 68-70. In Line 282-283 authors are discussing the possibility of difference in consumption values. As for example Timor-Leste animals population was 2 million in 2015 (**[**https://timor-leste.unfpa.org/sites/default/files/pub-pdf/2015%20Census%20Agriculture%20and%20Fisheries%20Report.pdf**](https://timor-leste.unfpa.org/sites/default/files/pub-pdf/2015%20Census%20Agriculture%20and%20Fisheries%20Report.pdf)**) compared to 1,523 million, 1,407 million, 1,299 million of Pakistan in 2019, 2018, and 2017, respectively. Whereas Cameroon is a country with diverse wildlife compared to in Pakistan.**

• Line 284: Consider the use of “consumption” instead of “exposure”

**RESPONSE: Line 331-332 accepted and changed: “Exposure” changed to “Consumption”.**

**Response to Reviewer 2**

The strengths of the paper are its description of the quantities of HPCIAs imported into Pakistan, as well as of growth promoters (GPs) and feed additives over time, and the use of import data for this purpose. Although “To best of our knowledge, this is the first to describe quantities, consumptions, and details on exporting countries/companies of growth promotors in veterinary sector”, does this really matter. It’s a good start and deserves to have this element published.

**RESPONSE: We thank the reviewer for the positive comments.**

The demonstrated that approximately 805 tonnes of seven selected CIA-HtP (net weight of 240 AAI) were imported between 2017 and 2019, an average of 268 tonnes per year. Although the authors seem surprised that “this volume far exceeds the total yearly import volumes of veterinary antimicrobials in other lower- and middle-income countries” actually they are probably not surprising. India used 10,000 tons of colistin in animal feed annually until recently.

**RESPONSE: Thank you for this information. As the authors discussed, based on methodology – import volumes and adjustment over animals biomass – the results cannot be readily compared to other available data. Moreover, country level veterinary antimicrobial consumption data is not available for most of the LMICs including India.**

The weaknesses of the paper are numerous, and not summarized in the Limitations section.

**RESPONSE: We have revised the Limitations section based on the comments of this and Reviewer #1. See also our responses above.**

For example, different assumptions were made of the relative weight content of shipments of 7 High Priority Critically Important Antimicrobials (HPCIA) described as more than one (or multiple) AM, or where indeed no net weight was given. For AM growth promoters, all imported as finished products, estimates were made of the amount of active ingredient imported and net weights also calculated.

**RESPONSE: As mentioned in methodology gross weight is available for all the shipments, mandatory in the bill of lading, the authors developed a methodology to further refine the calculations for antimicrobial import volumes i.e., to eliminate the packaging weight. The percentage packaging weight (PPW) is calculated from the shipments with net weights available for the pharmaceutical raw material and applied to the rest of shipments with no net weight given in the product description. We are confident with our net weight calculations for rest of shipments as the exporting companies/manufacturers are the same as those for the shipments with net weights available.**

**For feed additives/growth promoters net weights are calculated as for the pharmaceutical raw materials. It is important to note, that no assumptions were made for the calculation of active ingredient (active chemical compound) for FA/GP products. The FA/GP product composition is used to calculate the active chemical compound.**

**The authors discussed in detail the limitations their possible description in “Limitations” section.**

The number and biomass of a wide variety of animals regarded as likely to be treated with AMs (buffaloes, cattle, goats, sheep, chickens, camels, asses, mules, and horses) and slaughtered for meat consumption were calculated from FAO data, and live weights estimated for these livestock from Pakistan or, if not available, from Asian generic sources. Pharmaceutical raw ingredients usage was calculated from total animal biomass and growth promoter active ingredient use by chicken biomass. Pakistan does not grow swine so that it is assumed probably reasonably that all growth promoters go to chickens. However, we really do not know where these AMs went and whether these animals are treated (or fed) with AMs, so that Figures 5 and 6 may be meaningless.

**RESPONSE: Biomass and antimicrobial consumption calculations were made using methodology published and used by the OIE** [**https://www.oie.int/app/uploads/2021/05/a-fifth-annual-report-amr.pdf**](https://www.oie.int/app/uploads/2021/05/a-fifth-annual-report-amr.pdf) **and** [**https://www.frontiersin.org/articles/10.3389/fvets.2019.00317/full**](https://www.frontiersin.org/articles/10.3389/fvets.2019.00317/full)**. For camels, conversion coefficient (K), for conversion of carcass weight to live weight, was taken form** [**https://www.cabi.org/bookshop/book/9781780641010/**](https://www.cabi.org/bookshop/book/9781780641010/) **as the OIE recommended manual did not hold the information on camels** [**https://circabc.europa.eu/sd/a/90447c6f-5b7c-4b6f-87e9-27c5a7a5c923/ASA-TE-F-655%2520SBS%2520Manual%2520-%2520meat.doc**](https://circabc.europa.eu/sd/a/90447c6f-5b7c-4b6f-87e9-27c5a7a5c923/ASA-TE-F-655%2520SBS%2520Manual%2520-%2520meat.doc)

**Antimicrobials in veterinary are used for prophylaxis, treatment, and growth promotion purpose. For prophylaxis and treatment antimicrobials can be injected or fed as for example in poultry all the medication either for prophylaxis or treatment is given orally either via water or feed. FAs/GPs are imported as granular products imported by feed manufacturers or feed ingredient importers whereas pharmaceutical raw material are pure chemical compounds in powdered form imported by pharmaceutical manufacturing companies. So, we have a demarcation among PRMs and FAs/GPs. All the PRM and FAs/GPs import volumes are adjusted for total animal biomass as per OIE’s methodology. However, limitations on the manufacturing and use of these antimicrobials are discussed in “Limitations” section. Figures 5 and 6 are Treemap-charts (**[**https://r-graph-gallery.com/treemap.html**](https://r-graph-gallery.com/treemap.html) **and** [**https://support.microsoft.com/en-us/office/create-a-treemap-chart-in-office-dfe86d28-a610-4ef5-9b30-362d5c624b68**](https://support.microsoft.com/en-us/office/create-a-treemap-chart-in-office-dfe86d28-a610-4ef5-9b30-362d5c624b68)**) used to visualize and compare proportions within a hierarchy instead of absolute values and hence, are useful.**

The authors described sampling on 10 dairy and 10 chicken farms, but never refer to this data again; it would have been useful, perhaps. In the absence of knowing how these AMs are used, the data are not helpful, especially adjusted to “biomass” metrics. There is no attempt to compare such metrics with other countries, which would have been interesting.

**RESPONSE: Surveillance on ten broiler and dairy farms was used as proxy to select the most frequently used antimicrobials in the veterinary sector. We do not refer back to it as it is only a justification for our sample selection of antibiotics.**

**The paper focuses on the national level veterinary consumption of CIA-HtP antimicrobials and not on the farm level usage OR how these antimicrobials are used as published previously (**[**https://pubmed.ncbi.nlm.nih.gov/31795863/**](https://pubmed.ncbi.nlm.nih.gov/31795863/) **;** [**https://pubmed.ncbi.nlm.nih.gov/33324699/**](https://pubmed.ncbi.nlm.nih.gov/33324699/) **;** [**https://pubmed.ncbi.nlm.nih.gov/34069928/**](https://pubmed.ncbi.nlm.nih.gov/34069928/)**). The objective of this paper is to quantify national level imports volumes of CIA-HtP and their consumption as per the OIE recommended methodology.**

**There is a lack country level data on veterinary consumption of antimicrobials from LMICs (https://www.mdpi.com/2079-6382/7/3/75) and countries with similar agricultural system as of Pakistan. Moreover, the difference in methodologies makes comparison difficult among the available data as mentioned in Lines 338-341 “It is difficult to compare the veterinary antimicrobial consumption per unit biomass due to lack of any standardized metric and monitoring system [30]. Moreover, our findings are difficult to be compared with other AMU studies because we focused only on critically important antimicrobials and not all classes of antimicrobials.”**

The different metrics used are confusing: for example, “805 tonnes of seven selected CIA-HtP (net weight of 240 AAI)

**RESPONSE: Line 278-280 correction made: “The study demonstrated that approximately 805 tonnes of seven selected CIA-HtP (net weight of PRM) were imported between 2017 and 2019, an average of 268 tonnes per year.”**

Minor comments

Figure 1: These are confusing since it says “different countries” but only shows China. I now see that the figure does show countries and indicates the relative contribution by the size of the circle; most are almost invisible. I’d delete the confusing Figure and just keep the data in the text. Figure 1b (not labelled as such) seems to show different Provinces in China, why not say this in the legend, and if this is correct does this matter? What is the difference from Figure S1?

**RESPONSE: Subsets “A. and B.” mentioned in Figures 1 and S1 and the legends changed as:**

**“Figure 1: Total volumes and ratios of pharmaceutical raw materials and feed additives/growth promoters imported from: A. different countries and B. China and its different provinces”**

**Figure 1 describes the import volumes of antimicrobials in terms of pharmaceutical raw material (PRM) or antimicrobial chemical compound (ACC) as for example colistin sulphate or tilmicosin phosphate whereas Figure S1 describes the total import volumes of antimicrobial product/substance shipped i.e., feed premix zinc bacitracin 15% (15 mg of ACC in 100 mg of product/substance). Figure S1 describes the actual amount/tonnage of FA/GP products shipped/imported in Pakistan irrespective of the amount of ACC each product contain.**

Figure 2: It’s hard to see the colours for norfloxacin and ceftiofur, in fact I don’t see any ceftiofur. Make a note in the legend (Table 2 shows a very small amount imported). What’s the difference in Figure 3 and Figure S2?

**RESPONSE: Figure 3 describes the import volumes of FAs/GPs in terms of antimicrobial chemical compound (ACC) each product contains as for example feed premix zinc bacitracin 15% (15 mg of ACC in 100 mg of product/substance). Whereas Figure S2 describes the total import volumes of FA/GP product/substance shipped/imported in Pakistan irrespective of the amount of ACC each product contain.**

**We agree with reviewer and added “For small amounts imported referred to Table 2”. in figure legends.**

The Figure legend for Figure 4 is very poor; there are no units given. Do you mean relative?

**RESPONSE: Legends added in Figures 4, 5, and 6.**

Table S2 should be given in the main paper, it’s important.

**RESPONSE: Table S2 is about total animal biomass of different species calculated from population census and with OIE reference methodology (1). Since Table S2 is very extensive, we decided not to include it in the main manuscript. We have given average animal biomass figure in main manuscript as Fig 4.**

Zinc bacitracin and BMD are the same drug essentially, bacitracin.

**RESPONSE: Zinc bacitracin and BMD are calculated as separate antimicrobial chemical compound (ACC) used as feed additive or growth promoter Table S4. Moreover, this is done to quantify separately the most used ACCs in the poultry feed.**

“During the three years a staggering 577 tonnes of antimicrobial active ingredients were imported as growth promoters, which is more than two thirds of the total CIA-HtP imported of this study”. However, the great majority of GPs are not CIA-Htp (HPCIAs), so it’s not clear what this statement means.

**RESPONSE: For Pakistan, during the three years studied, 577 tonnes of ACCs were imported as growth promoters, this indicates the FAs/GPs are still being used in food animals production despite their ban in several countries in Asia [24,25] and rest of the world [26,27].**
